# Introducing an online training course in Campbell systematic review methods

**DOI:** 10.1002/cl2.1300

**Published:** 2023-01-13

**Authors:** Jeffrey C. Valentine, Julia H. Littell, Sarah Young, Greg Bunyea

**Affiliations:** ^1^ University of Louisville Louisville KY USA; ^2^ Bryn Mawr College Philadepehia PA USA; ^3^ Carnegie Mellon University Pittsburgh PA USA; ^4^ Open Learning Initiative Pittsburgh PA USA

In April 2020, members of the Campbell Collaboration Methods Group and Campbell leadership met to discuss options for creating flexible training opportunities for Campbell reviewers. It was not a coincidence that this meeting occurred at the beginning of the Covid‐19 pandemic. But in truth, conversations about how Campbell might increase the effectiveness and reach of Campbell training started at least a decade earlier. Training in systematic review methods has always been important to Campbell—we have a Methods sub‐group that is focused on training, and training opportunities have been part of every Campbell annual meeting. In addition, Campbell typically offers one or two standalone workshops sessions a year to outside groups seeking training in systematic review methods. We have never had a good way of evaluating the effectiveness of these one‐off training experiences, and in addition, we worried about the cost and access issues associated with in‐person training. Further, we could not help but notice that when Campbell training sessions are accessible to a broad audience, they tend to be very popular. As an example of this latter point, David Wilson's presentation on effect sizes and basic issues in meta‐analysis, which was part of a training workshop at the Campbell Colloquium in 2011, has been viewed over 49,000 times (Wilson, 2011) as of December 2022.

As we investigated options for addressing questions regarding training effectiveness, resource efficiency, and access equity, we searched for platforms that would allow us to host training materials in an online environment, that could be accessed at little or no cost to users, and that have tools for assessing learning. Ultimately, we chose to work with the Open Learning Initiative (OLI) at Carnegie Mellon University (https://oli.cmu.edu/). Over the course of the next 30 months, a team of seven individuals with expertise in systematic reviews and meta‐analysis, led by Jeff Valentine, Julia Littell, and Sarah Young, plus Greg Bunyea, a learning engineer from OLI, devoted thousands of hours to create a course titled: *Systematic reviews and meta‐analysis: A Campbell Collaboration online course* (Valentine et al., 2022). We were ably assisted in this work by Mark Englebert, Jennifer Hanratty, Terri Pigott, and Zahra Premji. The remainder of this essay is devoted to describing the scope of the course, its primary audience, its organization, and the principles we adopted during development.

## 1 COURSE PURPOSE AND ORGANIZATION

*Systematic reviews and meta‐analysis: A Campbell Collaboration online course* is aimed at Campbell reviewers and others who want to learn how to find, assess, and synthesize the results of relevant studies to inform policy, practice, and future research or in other words, people who want to learn how to conduct systematic reviews and meta‐analysis. We assume that learners will have prior graduate training in research methodology and statistics. We designed the course to be suitable for both classroom and independent learning and view it as the equivalent to a textbook or an introductory, graduate‐level course in systematic reviewing. It should also work well as an adjunct to in‐person workshop training. The content on systematic review methods is relevant to systematic reviews regardless of the nature of the specific research hypotheses being investigated, but the content addressing synthesis methods is focused on the synthesis of quantitative data (meta‐analysis).

## 2 COURSE CONTENT

The course is organized into eight Units that follow the steps in the review process:

1. Introduction
2. Problem formulation
3. Searching the literature
4. Screening potentially eligible studies
5. Data extraction and coding
6. Introduction to effect sizes
7. Introduction to meta‐analysis
8. Completing systematic reviews and exploring other synthesis methods

Units are the primary organizing framework, and each Unit contains multiple learning modules. For example, Unit 3 Searching the Literature has modules on the importance of working with an information specialist, how to identify sources to search, how to design database searches, and how to search the grey literature, among others. Most modules have multiple pages that serve to break the material into smaller chunks. For example, the module on Designing Database Searches has pages on combining terms and concepts, using subject headings, and on the role of database limiters, among others. In alignment with our design principles, described below, most pages begin with specific learning objectives and end with formative assessment exercises. Student performance on these formative assessment exercises provides critical information about how well the materials are leading students to successfully meet the learning objectives. This feedback will support continual improvement of the course by allowing us to identify where we have been more and less effective.

## 3 DESIGN PRINCIPLES

When we set out to design the course, we committed to a set of principles informed by research and theory regarding how humans learn. We used an outcome‐driven curriculum design method known as “backwards design” (Richards, 2013; Wiggins & McTighe, 2005). This method involves beginning by articulating learning objectives, then determining how we will assess if the learning objectives have been met, and only then creating content. By starting with learning outcomes and intentionally designing curriculum around those outcomes, the course content and assessments are aligned in helping learners achieve the learning goals. We also employ principles of active learning by providing opportunities for practice and formative assessments to test knowledge throughout the course (Koedinger et al., 2015). The practice and assessment activities include meaningful feedback, and in some cases hints, that challenges the learner to think critically about their answers. All assessments are linked in the OLI system to learning outcomes and skills. Thus, a well‐designed formative assessment can tell us something about how students are understanding or misunderstanding material, which we can then address through iterations on the course content.

## 4 NEXT STEPS

We announced the availability of a pilot version of this course (https://oli.cmu.edu/courses/systematic-reviews-and-meta-analysis/) at the What Works Global Summit in October 2022. We plan on releasing the full course in the early part of 2023. After launch, we will continue to make data‐driven improvements in course content and assessments. In the future, we intend to create summative assessments for self‐paced and classroom use, and we will explore the feasibility of expanding the course into a certificate program.
